# Association of Leukocyte Telomere Length with Fatigue in Nondisabled Older Adults

**DOI:** 10.1155/2014/403253

**Published:** 2014-02-16

**Authors:** Laila Bendix, Mikael Thinggaard, Masayuki Kimura, Abraham Aviv, Kaare Christensen, Merete Osler, Kirsten Avlund

**Affiliations:** ^1^Danish Aging Research Center, Universities of Southern Denmark, Aarhus, and Copenhagen, Denmark; ^2^Section of Social Medicine, Department of Public Health, University of Copenhagen, P.O. Box 2099, 1014 Copenhagen K, Denmark; ^3^Epidemiology, Institute of Public Health, University of Southern Denmark, JB Winslowsvej 9B, 5000 Odense C, Denmark; ^4^The Center of Human Development and Aging, University of Medicine and Dentistry of New Jersey, New Jersey Medical School, Newark, NJ 07103, USA; ^5^The Danish Twin Registry, Institute of Public Health, University of Southern Denmark, JB Winslowsvej 9B, 5000 Odense C, Denmark; ^6^Department of Clinical Genetics and Department of Biochemistry and Pharmacology, Odense University Hospital, 5000 Odense C, Denmark; ^7^Center for Healthy Aging, University of Copenhagen, 2200 Copenhagen N, Denmark; ^8^Research Center for Prevention and Health, The Capital Region of Denmark, 2600 Glostrup, Denmark

## Abstract

*Introduction*. Fatigue is often present in older adults with no identified underlying cause. The accruing burden of oxidative stress and inflammation might be underlying factors of fatigue. We therefore hypothesized that leukocyte telomere length (LTL) is relatively short in older adults who experience fatigue. *Materials and Methods*. We assessed 439 older nondisabled Danish twins. LTL was measured using Southern blots of terminal restriction fragments. Fatigue was measured by the Mob-T Scale based on questions on whether the respondents felt fatigued after performing six mobility items. *Results*. LTL was significantly associated with fatigue (*P* = 0.023), showing an increase of 0.038 kb/fatigue score unit. Aging-related diseases and mental health did not explain the association, while lifestyle factors slightly attenuated the estimates. *Conclusion*. Our results support an association between LTL and fatigue. Further studies are required to confirm this finding and the link of LTL with oxidative stress/inflammation over the life course.

## 1. Introduction

Fatigue is a common complaint among older adults. While it is often a symptom of psychiatric or medical illness, for many older persons, it is not possible to identify the underlying cause. Studies during the last 15 years have consistently shown that fatigue in older adults is a strong predictor of functional limitations [1], disability [2], and mortality [3]. Thus, fatigue might represent an early stage of frailty, defined as a physiologic state of increased vulnerability to stressors that results from decreased reserves in multiple physiologic and biologic systems [4].

Cellular and organismal damages caused by an accruing burden of oxidative stress could be an underlying factor to fatigue. Increased oxidative stress can be caused by defective mitochondria as well as by chronic low-grade inflammation. Both these conditions are known to be associated with increasing age, and evidence suggests that oxidative stress and inflammation are associated with frailty [5].

It is generally accepted that oxidative stress and inflammation are important contributors to the pathology of aging-related diseases such as cardiovascular disease [6], dementia [7], and cancer [8]. Such conditions often predispose to frailty [4]. Leukocyte telomere length (LTL) ostensibly undergoes an accelerated shortening due to inflammation and increased oxidative stress [6, 9]. This might explain the association of shortened LTL with aging-related diseases and some of their risk factors, including coronary heart disease, dementia, and insulin resistance [10, 11].

Potentially, shortened LTL and the consequent cellular aging could thus be an underlying mechanism to fatigue.

Woo et al. [12] found no apparent association between LTL and frailty in a study that applied a composite frailty index score but had not examined directly the potential connection between LTL and fatigue. To examine whether a link in fact exists between LTL and fatigue, we have exploited the same-sex twin design that has previously made it possible to show relationsships of LTL with mortality [13], and physical ability [14]. In the present study we show results supporting the association between LTL and fatigue.

## 2. Material and Methods

### 2.1. Description of Twin Sample

The Longitudinal Study of Aging Danish Twins (LSADT) began in 1995 with an assessment of same-sex twin pairs born in Denmark before 1920 [15]. Since 1995 twins from the LSADT have participated in a comprehensive face-to-face interview including detailed questions about health, lifestyle, and physical functioning [16]. This interview has been repeated every second year for a total of six examinations. At the second examination, blood samples were collected from 283 twin pairs. Of these, a total of 274 pairs (548 individuals) had their LTL measured [13].

For this study, the population was restricted to the 439 individuals who were nondisabled, that is, who were able to rise from a chair or bed, walk indoors, get outdoors, walk outdoors in nice weather, walk outdoors in bad weather, and climb stairs without help. Further, we excluded persons who had answered the questionnaire by a proxy. These restrictions were applied as previous studies have shown fatigue to be related to onset of disability [2], functional limitations [1], and even cardiovascular disease [17] among nondisabled and nondiseased. We also repeat the analyses in a restricted sample of persons below 80 years, as these persons are more likely to be without conditions, which we have not been able to adjust for.

For further information on the participants, see Table 1.

The study has been approved by the local scientific-ethical Committee. All participants provided written informed consent.

### 2.2. LTL Measurement

Leukocyte DNA was extracted using standard techniques. DNA quality was assessed by electrophoresis. LTL was measured using Southern blots of the terminal restriction fragments after digestion with HphI and MnlI restriction enzymes; for 377 of the nondisabled twins the terminal restriction fragments were also generated by HinfI and RsaI digestion [18]. The HphI/MnlI products include predominantly true telomeric repeats, whereas the HinfI/RsaI product includes a proximal telomere segment that is not strictly TTAGGG. The HphI/MnlI products were on average 1.1 kb shorter than the HinfI/RsaI products, and the two LTL measures correlated strongly (*r* = 0.88, *P* < 0.001).

Samples from the cotwins in each twin pair were resolved in adjacent lanes of the same gel. Running DNA digests from the two cotwins in adjacent lanes on the same gel diminished the methodological variation within twin-pairs. In between-pair analysis the “gel effect” was included as covariate.

Each sample was measured in duplicate (performed on different gels) and the mean of the two samples was used. The interassay coefficient of variation was 2.5% for the HinfI/RsaI digest and 3.4% for the HphI/MnlI digest.

We present data from the HphI/MnlI digest, while results obtained with HinfI/RsaI digest can be found in the corresponding Supplementary (S) Material (see tables in the Supplementary Material available online at http://dx.doi.org/10.1155/2014/403253).

### 2.3. Fatigue

Fatigue was measured by the Mob-T Scale based on questions about six mobility items ((a) rising from a chair or bed, (b) walking indoors, (c) getting outdoors, (d) walking outdoors in nice weather, (e) walking outdoors in bad weather, and (f) climbing stairs). Every activity was described relative to whether the respondents did or did not feel fatigued afterwards. The mobility-tiredness Scale (Mob-T) counts the number of activities managed without fatigue. The highest scale value thus describes no fatigue. The construct validity of the scale was tested by the Rasch model for item analysis, which showed that the scale had satisfactory homogeneity [19, 20]. The data have recently been reanalyzed using a new test based on the Rasch model on the assumption of equal item discrimination—with satisfactory results [21]. Reliability tests showed agreement from 94.3% to 98.3% and kappa values from 0.72 to 0.88 for the included items on intrarater and interrater tests [22]. As this measure of fatigue focuses on fatigue in performing certain activities it is likely that it reflects the physiologic and biologic consequences of doing these activities.

### 2.4. Covariates

Covariates of lifestyle, mental health, and aging-related somatic diseases were included as potential confounding factors since previous studies have shown that they might be associated with fatigue and LTL [10, 23–29].

#### 2.4.1. Lifestyle

Smoking status was divided in never, former, and current smokers. BMI was deduced from self-reported height and weight. Physical activity was scored on a five-point scale deducted from self-reported level and frequency of physical activity. The answers were scored as follows: 0: “no physical activity,” 1: “seldom low intensity physical activity,” 2: “frequent low intensity physical activity,” 3: “seldom high and frequent low intensity physical activity,” and 4: “frequent high intensity physical activity.”

#### 2.4.2. Mental Health Status

Cognitive performance was scored by a cognitive composite score. This was evaluated by a battery of five brief cognitive tests, which were selected to be sensitive to age-related changes. The tasks included a verbal fluency test, forward and backward digit span, and immediate and delayed recall of a 12-item list. The standard scores for each task were summed to give an overall score. Higher cognitive composite scores indicated better cognitive function [30].

Depression symptomatology was assessed using an adaptation of the depression section of the Cambridge Mental Disorders of the Elderly Examination (CAMDEX) [31]. McGue and Christensen [15] factor analyzed the LSADT depression items identifying two factor scales (9-item affective and 8-item somatic scales). Because these scales were highly correlated (*r* > 0.50), a 17-item total scale score has been defined [15]. Analyses reported here are based on the total scale score.

#### 2.4.3. Somatic Disease

Cardiovascular disease included angina pectoris, heart failure, hypertension, irregular heart rhythm, and myocardial infarction. For diabetes and cancer any positive history is regarded as being a positive outcome and no distinction is done between type and duration. Rheumatologic disease covers the presence of either of the following conditions: rheumatic arthritis, osteoarthritis, and/or gout.

#### 2.4.4. Statistical Methods

We applied a multiple regression modeling to test if fatigue was associated with LTL. A random intercept regression model was considered to account for nonindependence within pairs [32].

Possible association was further studied using an intrapair twin design in which difference in LTL was regressed on the difference in fatigue. This design enables us to control for age, sex, zygosity, and gel effect, as well as for early common environment and genetic factors. MZ twins share 100% of their genes while DZ twins only share 50%. If an association is attenuated among MZ twins it suggests genetic confounding. If an association is attenuated among MZ and DZ twins it suggests early environmental confounding.

A *P* value of 0.05 was chosen as significance level for all tests and the statistical analyses were done using STATA 11.1 (STATA Corp, College Station, TX, USA).

## 3. Results

### 3.1. General Characteristics

A total of 439 twins representing 257 twin pairs were studied. 145 (33%) were men, and 294 (67%) were women; 235 (53.5%) came from a dizygotic twin pair (DZ) and 204 (46.5%) from a monozygotic twin pair (MZ). Descriptive statistics of participants are presented in Table 1. The mean ages of the participants were 78.1 years for men (range 73–88) and 78.5 years for women (range 73–94).

Women were found to have longer LTL than men (age adjusted: 4.65 kb and 4.46 kb, resp.). The age-related attrition in this population was 0.020 kb per year. There was no significant difference in fatigue score between men and women. Fatigue was inversely correlated with age (*R* = −0.25, *P* < 0.001), that is, the higher the age the more fatigued (lower score).

The association between the examined covariates and, respectively, LTL and fatigue is presented in Table 2. Only smoking and depression symptomatology were significantly associated with LTL, while BMI, physical activity, cognitive function, depression symptomatology, and rheumatic disease were significantly associated with fatigue.

### 3.2. Multivariate Analysis

The regression model revealed a positive association between LTL and fatigue when adjusting for effects of sex and age. For every unit increase in fatigue score, LTL increased by 0.038 kb after adjustment for sex and age (*P* = 0.023; Table 2), which is equivalent to two years of age-related LTL attrition. There was no effect of zygosity nor was there an interaction effect of sex and age (data not shown) on the association. Hence, the association between fatigue and LTL was similar for each gender and zygosity.

The examined lifestyle factors had a minimal effect on the association between LTL and fatigue (Table 3, model 3), diminishing the association coefficient from 0.038 to 0.032. Including mental health in the model (Table 3, model 4) only slightly attenuated the association. However, inclusion of these covariates was not found to give a significantly better fit by a likelihood ratio test. Adjusting for somatic diseases separately or jointly did not influence the association between LTL and fatigue (Table 3, model 5).

Including all covariates, that is, sex, age, gel effect, zygosity, somatic diseases, mental health, and lifestyle resulted in slight attenuation of the association between LTL and fatigue (Table 3, model 6) (*P* = 0.166). However, likelihood ratio test indicated that the optimal model of the LTL-fatigue association includes only sex and age (model 2).

The analyses were repeated in a subsample of the study population restricted to individuals below 80 years. The LTL-fatigue association was found to be stronger in this younger subpopulation.

### 3.3. Intrapair Analysis

87 pairs were intact and discordant for the fatigue score. The intrapair analysis confirmed that for every one unit change in fatigue score, LTL differed by 0.039 kb (*P* = 0.054) within the pair (Table 4). This effect was not significantly different between MZ and DZ twin pairs; however, the effect only reached significance in MZ twin pairs.

The association was attenuated when including lifestyle factors from Coef = 0.039 to Coef = 0.030 due to a small confounding effect of smoking and physical activity not seen in the regression analysis. Likelihood ratio test suggested the inclusion of smoking in the model. Also inclusion of depression symptoms provided a better fit of the data but hardly influenced the association (Coef = 0.036, *P* = 0.097) (data not shown).

### 3.4. Results from HinfI/RsaI Digest

This subpopulation consisted of 377 individuals representing 219 twin pairs. There was no noteworthy difference on the demographics of two populations (Table S1). There were similar associations between the examined covariates and, respectively, LTL and fatigue (Table S2).

The association with fatigue was of lesser magnitude and no longer reached significance (Table S3, model 2), neither for the younger subpopulation. Likelihood ratio test showed that the inclusion of lifestyle factors (especially smoking) as well as depression symptomatology had a better fit of data. Including these factors completely attenuated the association (Coef = 0.005, *P* = 0.846).

The intrapair analyses were in agreement with findings from the HphI/MnlI digest, however not significant, and could mostly be attributed to physical activity and smoking, as well as depression symptomatology.

## 4. Discussion

In this study we observed an association between fatigue and LTL in nondisabled older twins. Previous studies have found fatigue to be related to onset of several adverse health outcomes [33], also when adjusted for multiple covariates. The predictive value of fatigue has been especially strong in nondisabled older persons and in persons without cardiovascular disease, indicating that fatigue may be an early marker of the aging process, before the onset of diseases and disability. We have earlier shown that LTL was related to physical ability, measured by a self-reported strength scale, in a study population of 70–93-year-old participants in the LSADT study [14]. The present study shows that LTL is associated with fatigue before the onset of disablement, thus suggesting that shortening of LTL is an early detectable event in this process.

Fatigue is a central symptom in many organic and psychiatric diseases. We have chosen to include four major aging-related diseases (CVD, diabetes, rheumatic disease, and cancer) as well as cognitive function as potential confounders since these have previously been suggested to be associated with LTL dynamics. However, we found no influence of either diseases or cognitive function on the LTL-fatigue association. Depression is a condition closely correlated with fatigue and is evidently associated with short LTL [34]. Thus, we considered depression a possible confounder. The inclusion of the available depression symptomatology score [15] slightly attenuated the association. It is likely that this finding is merely an over adjustment since the depression symptomatology score contains questions on feeling of lack of energy, which was highly correlated with fatigue (*R* = 0.323, *P* < 0.001). The coexistence of fatigue and depression has previously been reported [35] and found to be associated with an increased risk of death in older adults [36], as has the coexistence of depression and other diseases predisposing to fatigue. A common inflammatory and oxidative stress pathway has been suggested [37], necessitating a clarification of this coexistence in future studies.

Of the included lifestyle factors, we found that only smoking had a slight effect on the association between LTL and fatigue. We have previously found that physical activity level was associated with LTL [14]. In this subpopulation the association was of the same magnitude but not significant. We suggest that this is simply due to lack of power and therefore chose to include physical activity as a possible confounder. However, physical activity seemed only to have an effect on the association when LTL was measured with the HInfI/RsaI digest. Smoking is therefore the only of the analyzed covariates that we suggest as a confounder to be included in the model besides sex and age.

A study by Woo et al. [12] found no association between frailty and LTL. The discrepancy is likely due to the focus on fatigue in the present study, while Woo and colleagues used a composite frailty index. Methodological variation could also have an impact, as Woo et al. reported a CV of 11.1% in their qPCR based method, while the CV of the TRF assay for this cohort was only 3.4%. Moreover, although our cohort was of limited size, we gained statistical power by exploiting the twin design.

Some limitations and strengths should be considered when interpreting the results. The low coefficient of variation is a strength to our study, as well as the twin design. We have included lifestyle factors and major aging-related diseases as covariates; in future studies inclusion of medications could be of value since these could contribute to fatigue. Various tools to evaluate fatigue exist [38]. Many scales have been suggested, some focusing on the severity of fatigue. Further different performance based measures are often used (e.g., an oxygen uptake measure or accelerometers) [39]. The used fatigue measure is reliable [1, 22] and has the advantage that it is a self-reported measure of fatigue in performing certain activities and is therefore likely to reflect the physiologic and biologic consequences of doing these activities [33]. Our study suggests a possible common physiological pathway between LTL attrition and fatigue; however, to establish a better insight into the dynamics in fatigue and cellular aging, future studies should examine changes over time of LTL and fatigue score in the same individuals.

## 5. Conclusion

Overall, our findings indicate that LTL is associated with fatigue and that this cannot be explained by common lifestyle factors, aging-related diseases, or cognitive function. These findings largely agree with previous studies showing that fatigue is a strong indicator of adverse health outcomes in nondisabled older adults [33] and in middle-aged healthy men [17]. The findings are suggestive of oxidative stress and inflammation being underlying factors of fatigue and should spur the search for more biomarkers leading to a better understanding of the physiology behind fatigue.

## Supplementary Material

S-table 1-4 present data from the results obtained with HinfI/RsaI digest for which the inter-assay coefficient of variation was 3.4%.

## Figures and Tables

**Table 1:** Descriptive statistics of the study population, including data on mean LTL, fatigue and lifestyle, mental health and somatic disease covariates.

Characteristics		Total (*N* = 439)	Female (*N* = 294)	Male (*N* = 145)	*P* value (sex diff.)
LTL (in kb)	Mean (SD)	4.59	4.67	4.47	0.005*
Fatigue score	Mean (SD)	5.35	5.29	5.46	0.170
0	*N* (%)	4 (0.9%)	4 (1.4%)	0 (0.0%)	
1	*N* (%)	3 (0.7%)	3 (1.0%)	0 (0.0%)	
2	*N* (%)	17 (3.9%)	11 (3.7%)	6 (4.1%)	
3	*N* (%)	21 (4.8%)	15 (5.1%)	6 (4.1%)	
4	*N* (%)	29 (6.6%)	19 (6.5%)	10 (6.9%)	
5	*N* (%)	58 (13.2%)	42 (14.3%)	16 (11.0%)	
6	*N* (%)	307 (69.9%)	200 (68.0%)	107 (73.8%)	
Age	Mean(SD)	78.4	78.5	78.1	0.334
Zygosity	MZ *N* (%)	204 (46.5%)	138 (46.9%)	66 (45.5%)	
	DZ *N* (%)	235 (53.5%)	156 (53.1%)	79 (54.5%)	

Smoking	Never *N* (%)	154 (35.1%)	129 (43.9%)	25 (17.2%)	
	Former *N* (%)	155 (35.3%)	86 (29.3%)	69 (47.6%)	
	Current *N* (%)	139 (29.6%)	79 (26.9%)	51 (35.2%)	
BMI	Mean (SD)	24.13	23.65	25.09	<0.001*
<18.5	*N* (%)	25 (5.7%)	23 (7.8%)	2 (1.4%)	
18.5–24.9	*N* (%)	236 (53.8%)	174 (59.2%)	62 (42.8%)	
25–29.9	*N* (%)	157 (35.8%)	80 (27.2%)	77 (53.1%)	
>30	*N* (%)	17(3.9%)	13 (4.4%)	4 (2.8%)	
Physical activity	Mean (SD)	2.52	2.52	2.52	0.979
0	*N* (%)	62 (14.1%)	43 (14.6%)	19 (13.1%)	
1	*N* (%)	14 (3.2%)	9 (3.1%)	5 (3.4%)	
2	*N* (%)	152 (34.6%)	98 (33.3%)	54 (37.2%)	
3	*N* (%)	55 (12.9%)	40 (13.6%)	15 (10.3%)	
4	*N* (%)	156 (35.5%)	104 (35.4%)	52 (35.8%)	

Cognitive comp.	Mean (SD)	1.46	1.63	1.11	0.113
Depression sympt.	Mean (SD)	20.76	20.87	20.54	0.434

Rheumatic disease	No *N*	283	172	115	
	Yes *N* (%)	156 (35.5%)	122 (41.5%)	34 (23.4%)	
CVD	No *N*	280	182	98	
	Yes *N* (%)	159 (36.2%)	112 (38.1%)	47 (32.4%)	
Cancer	No *N*	389	258	131	
	Yes *N* (%)	50 (11.4%)	36 (12.2%)	14 (9.7%)	
Diabetes	No *N*	419	280	139	
	Yes *N* (%)	20 (4.6%)	14 (4.8%)	6 (4.1%)	

*Significant difference between sexes. ^*τ*^The *P* value for sex-differences has been adjusted for age.

LTL: leukocyte telomere length; CVD: cardiovascular disease; MZ: monozygotic twins; DZ: dizygotic twins.

**Table 2:** The association between covariates and, respectively, LTL, and fatigue.

Covariates	Association with LTL						Association fatigue					
	Raw			Adjusted (sex, age)			Raw			Adjusted (sex, age)		
	Coef^†^	CI (95%)	*P*	Coef^†^	CI (95%)	*P*	Coef^†^	CI (95%)	*P*	Coef^†^	CI (95%)	*P*
Fatigue (Score 0–6)	0.039	0.006–0.071	0.019	**0.038** ^††^	** 0.005–0.070**	**0.023**						
Age	−0.019	−0.035–−0.003	0.018	**−0.20**	−**0.036–**−**0.004**	**0.012**	−0.041	−0.073–−0.009	0.012	−**0.040**	−**0.072–**−**0.008**	**0.014**
Sex												
Male	Ref			Ref			Ref			Ref		
Female	0.161	0.032–0.290	0.014	** 0.169**	** 0.041–0.297**	**0.009**	−0.169	−0.426–0.088	0.197	−0.154	−0.408–0.099	0.233
Zygosity												
MZ	Ref			Ref			Ref			Ref		
DZ	−0.027	−0.150–0.097	0.672	−0.039	−0.159–0.081	0.530	−0.020	−0.263–0.223	0.871	−0.047	−0.286–0.192	0.700
Geleffect	0.007	0.000–0.014	0.040	0.002	−0.008–0.011	0.688						

Smoking												
Never	Ref			Ref			Ref			Ref		
Former	−0.076	−0.172–0.021	0.124	−0.063	−0.160–0.035	0.206	−0.132	−0.408–0.144	0.349	−0.208	−0.490–0.074	0.148
Current	−0.127	−0.234–−0.020	0.020	**−0.122**	**−0.230–−0.014**	**0.027**	−0.156	−0.448–0.135	0.293	−0.252	−0.547–0.043	0.095
Physical activity (Score 0–4)	0.024	−0.006–0.054	0.122	0.020	−0.011–0.049	0.213	0.344	0.266–0.423	<0.001	** 0.336**	** 0.257–0.415**	**<0.001**
BMI	−0.010	−0.022–0.003	0.137	−0.010	−0.023–0.003	0.127	−0.039	−0.072–−0.006	0.019	**−0.052**	**−0.085–−0.018**	**0.002**

Cognitive function	0.014	−0.000–0.027	0.053	0.011	−0.009–0.025	0.111	0.041	0.005–0.077	0.025	** 0.038**	** 0.002–0.074**	**0.037**
Depression symptomatology	−0.012	−0.022–−0.002	0.022	**−0.012**	**−0.022–−0.002**	**0.024**	−0.120	−0.145–−0.094	<0.001	**−0.118**	**−0.143–−0.093**	**<0.001**

Cancer												
No	Ref			Ref			Ref			Ref		
Yes	−0.005	−0.122–0.113	0.940	−0.010	−0.127–0.107	0.868	−0.230	−0.588–0.129	0.209	−0.236	−0.592–0.120	0.194
CVD												
No	Ref			Ref			Ref			Ref		
Yes	−0.009	0.095–0.076	0.829	−0.013	−0.098–0.072	0.763	−0.173	−0.414–0.068	0.160	−0.167	−0.406–0.072	0.171
Diabetes												
No	Ref			Ref			Ref			Ref		
Yes	0.001	−0.191–0.192	0.997	0.006	−0.184–0.196	0.952	−0.297	−0.852–0.257	0.293	−0.266	−0.817–0.284	0.343
Rheumatic												
No	Ref			Ref			Ref			Ref		
Yes	0.004	−0.080–0.087	0.933	−0.011	−0.095–0.072	0.788	−0.414	−0.656–−0.179	0.001	**−0.419**	**−0.659–−0.179**	**<0.001**

Bold values mark the significant adjusted coeffients.

^†^The coefficients shows the magnitude of the linear association between covariates and LTL respectively fatigue. First columns are raw data, while second columns are adjusted for sex and age.

^††^There is a significant association between LTL and fatigue when adjusting for age and sex. The coefficient of 0.038 tells that for every increase on the fatigue score (i.e. the less fatigued) the LTL is 0.038 kb longer.

**Table 3:** The association between LTL and fatigue.

Population		Model 1 Raw	Model 2 Age, sex	Model 3 Lifestyle	Model 4 Mental	Model 5 Somatic	Model 6 Full
All *N* = 439	Coef	0.039	0.038	0.032	0.032	0.038	0.026
	CI 95%	0.006–0.071	0.005–0.070	−0.003–0.070	−0.002–0.067	0.005–0.071	−0.011–0.064
	*P*-value	0.019	0.023	0.074	0.066	0.025	0.166

<80 y *N* = 315	Coef	0.050	0.051	0.044	0.051	0.050	0.044
	CI 95%	0.007–0.093	0.008–0.094	−0.002–0.090	0.003–0.099	0.006–0.094	−0.007–0.095
	*P*-value	0.024	0.019	0.058	0.037	0.025	0.093

Model 1: raw model.

Model 2: adjusted for sex and age.

Model 3: as Model 2 including adjustment for lifestyle factors.

Model 4: as Model 2 including adjustment for mental health.

Model 5: as Model 2 including adjustment for somatic disease.

Model 6: full model that is as Model 2 including adjustment for lifestyle factors, mental health, somatic disease as well as zygosity and gel-effect.

**Table 4:** Intrapair analysis.

		All	MZ	DZ
All *N* = 174	Coef	0.039	0.057	0.026
	CI 95%	−0.001–0.079	0.007–0.107	−0.035–0.086
	*P*-value	0.054	0.027	0.398

<80 y *N* = 110	Coef	0.058	0.069	0.049
	CI 95%	0.003–0.112	0.002–0.138	−0.035–0.132
	*P*-value	0.040	0.049	0.247

The intrapair analysis is shown for the whole sample as well as for the younger subpopulation. Further the analysis has been run for only MZ and DZ twins respectively.

The simple model is intrinsically adjusted for sex, age, geleffect and zygosity due to the intrapair twin design.

LTL: leukocyte telomere length; MZ: monozygotic twins; DZ: dizygotic twins.
