# Supplementary material for: Association of Leukocyte Telomere Length with Fatigue in Nondisabled Older Adults
Source: J Aging Res. 2014 Feb 16;2014:403253. doi: 10.1155/2014/403253 (PMC3945148; doi:10.1155/2014/403253)
Supplement: Supplementary file 1 — S-table 1-4 present data from the results obtained with HinfI/RsaI digest for which the inter-assay coefficient of variation was 3.4%. [file 403253.f1.pdf]

Table S1 Descriptive statistics of the study population.

Characteristics		Total (N=377)	Female (N=252)	Male (N=125)	p-valueτ (sex diff)
LTL (in kb)	mean(SD)	5.73	5.80	5.61	0.006*
Fatigue score	mean(SD)	5.33	5.27	5.45	0.187
0	N (%)	4 (1.1%)	4 (1.6%)	0 (0%)	
1	N (%)	2 (0.5%)	2 (0.8%)	0 (0%)	
2	N (%)	15 (4.0%)	10 (4.0%)	5 (4.0%)	
3	N (%)	21 (5.6%)	15 (6.0%)	6 (4.8%)	
4	N (%)	25 (6.6%)	16 (6.3%)	9 (7.2%)	
5	N (%)	47 (12.5%)	34 (13.5%)	13 (10.4%)	
6	N (%)	263 (69.8%)	171 (67.8%)	92 (73.6%)	
Age	mean(SD)	78.8	78.9	78.5	0.277
Zygosity	MZ N(%)	174 (46.2%)	120 (47.6%)	54 (43.2%)	
	DZ N(%)	203 (53.8%)	132 (52.4%)	71 (56.8%)	
Smoking	Never N (%)	137 (36.3%)	113 (44.8%)	24 (19.2%)	
	Former N (%)	131 (34.7%)	74 (29.4%)	57 (45.6%)	
	Current N (%)	109 (28.9%)	65 (25.8%)	44 (35.2%)	
BMI	mean(SD)	24.07	23.58	25.05	<0.001*
Physical activity	mean(SD)	2.51	2.51	2.52	0.892
0	N (%)	53 (14.1%)	35 (13.9%)	18 (14.4%)	
1	N (%)	8 (2.1%)	6 (2.4%)	2 (1.6%)	
2	N (%)	139 (36.9%)	91 (36.1%)	48 (38.4%)	
3	N (%)	47 (12.5%)	36 (14.3%)	11 (8.8%)	
4	N (%)	130 (34.5%)	84 (33.3%)	46 (36.8%)	

Characteristics continued.			Total (N=377)	Female (N=252)	Male (N=125)	p-value (sex diff)
Cognitive comp.	mean(SD)		1.50	1.70	1.09	0.058
Depression sympt.	mean(SD)		20.9	20.9	20.9	0.932
Rheumatic disease	No	N	247	150	97	
	Yes	N(%)	130 (34.5%)	102 (40.5%)	28 (22.4%)	
CVD	No	N	238	153	85	
	Yes	N(%)	139 (36.9%)	99 (39.3%)	40 (32.0%)	
Cancer	No	N	338	221	117	
	Yes	N(%)	39 (10.3%)	31 (12.3%)	8 (6.4%)	
Diabetes	No	N	360	240	120	
	Yes	N(%)	17 (4.5%)	12 (4.8%)	5 (4.0%)	

Table S2. The association between covariates and LTL respectively fatigue.

Covariates		Association to LTL						Association to fatigue					
		Raw			Adjusted (sex, age)			Raw			Adjusted (sex, age)		
		Coef[†]	CI (95%)	p	Coef[†]	CI (95%)	p	Coef[†]	CI (95%)	p	Coef[†]	CI (95%)	p
Fatigue (Score 0 – 6)		0.029	-0.008 – 0.065	0.121	0.027[††]	-0.009 – 0.063	0.140						
Age		-0.021	-0.039 – -0.003	0.023	**-0.022**	**-0.040 – -0.004**	**0.015**	-0.043	-0.078 – -0.008	0.017	**-0.042**	**-0.077 – -0.007**	**0.019**
Sex	Male	Ref			Ref			Ref			Ref		
	Female	0.173	0.024 – 0.323	0.023	**0.183**	**0.035 – 0.331**	**0.015**	-0.180	-0.466 – 0.107	0.219	-0.161	-0.444 – 0.122	0.264
Zygosity	MZ	Ref			Ref			Ref			Ref		
	DZ	-0.062	-0.205 – 0.081	0.394	-0.083	-0.225 – 0.016	0.251	0.001	-0.271 – 0.272	0.996	-0.044	-0.312 – 0.224	0.748
Gel-effect		0.010	0.001 – 0.019	0.023	0.005	-0.007 – 0.016	0.415						
Smoking	Never	Ref			Ref			Ref			Ref		
	Former	-0.101	-0.208 – 0.007	0.066	-0.089	-0.198 – 0.183	0.103	-0.204	-0.507 – 0.098	0.185	-0.274	-0.580 – 0.032	0.079
	Current	-0.166	-0.285 – -0.046	0.007	**-0.163**	**-0.283 – 0.043**	**0.008**	-0.221	-0.541 – 0.099	0.176	-0.310	-0.633 – 0.013	0.060
Physical activity (Score 0 – 4)		0.043	0.008 – 0.077	0.016	**0.038**	**0.003 – 0.072**	**0.034**	0.341	0.252 – 0.429	<0.001	**0.331**	**0.241 – 0.420**	**<0.001**
BMI		-0.007	-0.022 – 0.007	0.328	-0.008	-0.022 – 0.007	0.309	-0.035	-0.072 – 0.001	0.060	**-0.050**	**-0.008 – -0.013**	**0.009**
Cognitive function		0.020	0.004 – 0.035	0.014	**0.017**	**0.001 – 0.032**	**0.037**	0.047	0.008 – 0.087	0.020	**0.044**	**0.004 – 0.083**	**0.032**
Depression symptomatology		-0.015	-0.026 – -0.003	0.012	**-0.014**	**-0.025 – -0.003**	**0.016**	-0.117	-0.145 – -0.090	<0.001	**-0.116**	**-0.144 – -0.089**	**<0.001**
Cancer	No	Ref			Ref			Ref			Ref		
	Yes	-0.042	-0.182 – 0.098	0.557	-0.051	-0.190 – 0.088	0.475	-0.287	-0.698 – 0.123	0.170	-0.270	-0.679 – 0.139	0.196
CVD	No	Ref			Ref			Ref			Ref		
	Yes	-0.019	-0.115 – 0.078	0.705	-0.027	-0.122 – 0.069	0.586	-0.187	-0.451 – 0.078	0.167	-0.186	-0.448 – 0.077	0.166
Diabetes	No	Ref			Ref			Ref			Ref		
	Yes	-0.007	-0.229 – 0.215	0.952	-0.010	-0.231 – 0.210	0.926	-0.114	-0.731 – 0.503	0.718	-0.098	-0.709 – 0.513	0.754
Rheumatic	No	Ref			Ref			Ref			Ref		
	Yes	0.028	-0.066 – 0.123	0.561	0.012	-0.082 – 0.107	0.800	-0.440	-0.704 – -0.176	0.001	**-0.437**	**-0.702 – -0.172**	**<0.001**

Table S3. The association between LTL and fatigue.

Population		Model 1 Raw	Model 2 Age, sex	Model 3 Lifestyle	Model 4 Mental	Model 5 Somatic	Model 6 Full
All N=377	Coef	0.029	0.027	0.011	0.016	0.027	0.0001
	CI 95%	-0.008 – 0.065	-0.009 – 0.063	-0.027 – 0.050	-0.022 – 0.055	-0.010 – 0.064	-0.041 – 0.041
	p-value	0.121	0.140	0.573	0.400	0.149	0.997
<80y N=257	Coef	0.044	0.046	0.030	0.040	0.041	0.023
	CI 95%	-0.005 – 0.093	-0.003 – 0.095	-0.022 – 0.081	-0.015 – 0.096	-0.009 – 0.092	-0.035 – 0.081
	p-value	0.078	0.067	0.261	0.157	0.110	0.442

Table S4. Intrapair analysis.

		All	MZ	DZ
All N=148	Coef	0.021	0.038	0.008
	CI 95%	-0.024 – 0.066	-0.026 – 0.102	-0.056 – 0.072
	p-value	0.357	0.239	0.807
<80y N=86	Coef	0.052	0.062	0.043
	CI 95%	-0.011 – 0.115	-0.029 – 0.153	-0.047 – 0.134
	p-value	0.105	0.175	0.347
